# Companion Vector-Borne Pathogens and Associated Risk Factors in Apparently Healthy Pet Animals (Dogs and Cats) in Khukhot City Municipality, Pathum Thani Province, Thailand

**DOI:** 10.3390/pathogens12030391

**Published:** 2023-03-01

**Authors:** Nam Hung Luong, Ketsarin Kamyingkird, Nipa Thammasonthijarern, Jumnongjit Phasuk, Burin Nimsuphan, Khampee Pattanatanang, Wissanuwat Chimnoi, Chanya Kengradomkij, Nutsuda Klinkaew, Tawin Inpankaew

**Affiliations:** Department of Parasitology, Faculty of Veterinary Medicine, Kasetsart University, Bangkok 10900, Thailand

**Keywords:** companion vector-borne pathogens, apparently healthy, domesticated animals, Thailand

## Abstract

Pet animals (dogs and cats) can be infected with several companion vector-borne pathogens (CVBPs). Morbidity and mortality have been reported in pet animals due to CVBP infections. Pet animals living in close proximity to humans are able to transmit zoonotic pathogens. This study used molecular techniques to investigate the prevalence of CVBPs in apparently healthy pet animals (dogs and cats) from Khukhot City Municipality, Pathum Thani province, Thailand. In total, 210 blood samples were randomly collected from 95 dogs and 115 cats for the detection of seven companion vector-borne pathogens (*Anaplasma*, *Babesia*, *Bartonella*, *Ehrlichia*, *Hepatozoon*, *Mycoplasma,* and *Rickettsia*) using polymerase chain reaction. The results showed that 10.5% (22/210) of apparently healthy pet animals were infected with at least one pathogen, comprising 6 dogs (6.3% of all dogs tested) and 16 cats (13.9% of all cats tested). *Ehrlichia* (6.3%) was present only in dogs; furthermore, 1.1% of the dogs were positive for *Anaplasma*. There was one dog case co-infected with two pathogens (1.1%). In cats, *Mycoplasma* (9.6%) was the predominant CVBP, followed by *Rickettsia* (4.4%). The DNA sequences of all positive animals were 97–99% homologous to those found in the GenBank™ database for all CVBPs identified, namely *Ehrlichia canis*, *Anaplasma platys, Rickettsia felis*, *Mycoplasma haemofelis*, and *Candidatus* Mycoplasma haemominutum. Additionally, the risk of infection with CVBPs in pets was significantly associated with age, with young dogs more likely to be infected with CVBPs than adult dogs (OR 8.5, 95% CI 1.4–50.1, *p* = 0.006), while adult cats were more likely to be infected with CVBPs than young cats (OR 3.8, 95% CI 1.0–14.0, *p* = 0.038). The detection of CVBPs demonstrated the potential risk of infection that may occur in apparently healthy pet animals in Pathum Thani province. These results confirmed that apparently healthy pet animals may still be at risk of vector-borne infections and could maintain the infection cycle in pet populations. Furthermore, sampling a greater number of apparently healthy pet animals may disclose predictors of CVBP positivity in domesticated animals in this area.

## 1. Introduction

Companion vector-borne diseases (CVBDs) are illnesses that are transmitted to animals, including pets, by vectors such as ticks, fleas, mosquitoes, and sandflies. These diseases are a growing medical concern worldwide [1]. Pets, especially dogs and cats, can play a significant part in the transmission of CVBDs to humans due to the close and often shared living environments between pets and their owners. Additionally, socio-economic factors, such as poverty and poor living conditions, can increase the risk of the pet-to-human transmission of CVBDs [2]. Ticks and fleas are considered the main vectors carrying many companion vector-borne pathogens (CVBPs) and causing tick-borne and flea-borne diseases in pets [3]. Apicomplexan protozoans (*Babesia* and *Hepatozoon*) and Alphaproteobacteria (*Anaplasma* and *Ehrlichia*) are common CVBDs causing illness in dogs [4]. In cats, cat scratch disease is one of the most common CVBDs caused by *Bartonella* through bites or scratches from infected fleas or by direct contact with infected animals [5]. It is also typically transmitted to humans through scratches or bites from infected cats. *Rickettsia* and *Mycoplasma* can be transmitted to both dogs and cats [6], as well as being potentially transmitted to humans [7].

Ticks and fleas are arthropod parasites that can transmit the most severe CVBDs to animals and humans [8]. Research on tick-borne and flea-borne diseases has not yet been widely undertaken, particularly in developing nations. In Thailand, *Rhipicephalus sanguineus*, the brown dog tick, is the most prevalent arthropod parasite on dogs. Sometimes, it is detected on cats in Thailand’s countryside, where there are a lot of dogs and cats in close proximity. *Ctenocephalides felis* and *Ctenocephalides orientis* were reported as the most common flea species in dogs and cats in Thailand [9]. CVBPs play a significant role in transmitting diseases to dogs and cats, with fleas and ticks recognized as the main vectors. These vectors can carry various pathogens, including *Ehrlichia canis*, *Ricketssia felis*, *Babesia vogeli*, *Hepatozoon canis*, or *Mycoplasma haemofelis* and *Bartonella henselae* [10,11,12,13]. The documented prevalence of CVBPs in animals has varied in numerous epidemiological studies and has been continuously updated each year in Thailand [14,15,16]; nonetheless, there is limited research on CVBPs in companion animals, especially apparently healthy pet animals that are very close to humans. Several studies have recorded common CVBPs, including *Anaplasma* spp., *Babesia* spp., *Ehrlichia* spp., and *Hepatozoon* spp. [17,18,19]. Consequently, it is critical to concentrate on CVBP detection in pet animals (dogs and cats). Research investigating the epidemic’s current state and analyzing and comparing the genetic diversity of CVBPs discovered in dogs and cats should better control and prevent outbreaks of these diseases in these companion animals. This study intended to estimate the prevalence of CVBPs in apparently healthy Thai pet dogs and cats, as well as to investigate the risk factors related to CVBP infection in apparently healthy Thai pet dogs and cats.

## 2. Materials and Methods

### 2.1. Study Area and Sample Collection

**Figure 1:**
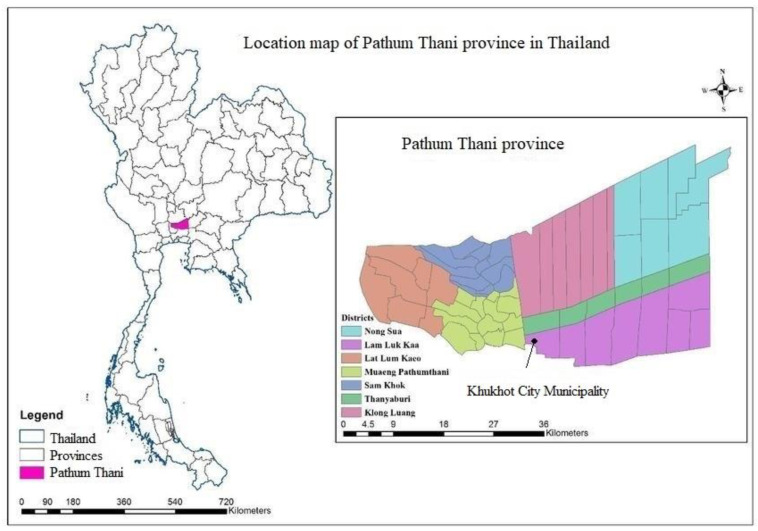
Map of study area in Pathum Thani province, Thailand. The inset shows the location of Khukhot City Municipality, where study samples were collected.

The appropriate sample size was determined for this study using the formula applied in a previous study [20], which considered a 95% confidence level (Z), 5% margin of error (e), and a sample proportion of approximately 5% (P) to account for an infinite population. Following the aforementioned calculation based on the prescribed formula, the sample determined for the current study was found to consist of 73 pet dogs and 73 pet cats. This sample size was deemed appropriate to ensure the desired level of confidence. Consequently, between June and August 2022, a total of 210 blood samples were randomly collected from domesticated dogs (*n* = 95) and cats (*n* = 115) in Pathum Thani province, Thailand (Figure 1). Blood samples were stored in an Ethylenediaminetetraacetic acid (EDTA) tube and kept in iceboxes (4–8 °C) before being transported within 2–4 h after collection to the laboratory at the Department of Parasitology, Faculty of Veterinary Medicine, Kasetsart University, Bangkok, Thailand for DNA extraction and the detection of CVBPs based on a molecular method. Sample collection was only completed after informed consent was granted from dog and cat owners. The pet caretakers or owners were interviewed by filling in a questionnaire regarding their animals. Data were documented on sex (male or female), age (≤1 year and >1 year), breed (pure or mixed), free roaming (yes or no), ectoparasite infestation (yes or no), and the tri-monthly application of ectoparasiticides (yes or no) for individual dogs and cats.

### 2.2. Molecular Detection of CVBPs in Dogs and Cats

Two hundred microliters of blood from each dog and cat sample was used for DNA extraction with a Genomic DNA Mini Kit (Geneaid Biotech Ltd., New Taipei City, Taiwan), according to the manufacturer’s instructions. Amplifications were performed in a 25 µL reaction mixture composed of 2.5 µL of 10× buffer; 10 pmol of each primer; 0.3 units of Taq DNA polymerase (Applied Biological Materials (ABM^®^) Inc., Richmond, BC, Canada); 2 µL of template DNA; and deionized distilled water. The primers and PCR protocols used in this study are shown in Table 1. Each PCR reaction was carried out using positive DNA extracted from blood infected with each of the 7 pathogens (*Ehrlichia* spp., *Anaplasma* spp., *Babesia* spp., *Hepatozoon* spp., *Mycoplasma* spp., *Bartonella* spp., and *Rickettsia* spp.), and negative control (nuclease-free water) PCR products were migrated in 1.5% agarose gel using electrophoresis and visualized under a UV transilluminator (Clare Chemical Research, Dolores, CO, USA). The positive samples were cut from the gel and purified using a gel and PCR purification kit (BioFACT^TM^, Daejeon, Republic of Korea), according to the manufacturer’s instructions, and then sequenced.

### 2.3. Sequence and Phylogenetic Analysis

The purified amplicons were analyzed using Sanger’s sequencing technology by a commercial company (Macrogen^®^, Seoul, Republic of Korea). The raw nucleotide sequences and chromatograms were viewed using BioEdit version 7.2 software (www.mbio.ncsu.edu/BioEdit/bioedit.html, accessed on 5 December 2022). The sequences were compared with published sequences using the basic local alignment search tool (BLAST) of the U.S. National Center for Biotechnology Information (https://blast.ncbi.nlm.nih.gov/Blast.cgi, accessed on 5 December 2022) to determine the *Anaplasma*, *Ehrlichia*, *Mycoplasma,* and *Rickettsia* pathogens/parasites. The maximum-likelihood analyses were conducted using Tamura–Nei parameter distance estimates, while the phylogenetic trees were constructed using Mega 6 software (www.megasoftware.net, accessed on 25 January 2023). Bootstrap analyses were conducted using 1000 replicates, and the sequence of *Ancylostoma caninum* was used as the outgroup.

### 2.4. Statistical Analysis

Statistical analysis was conducted using R software version 4.0.5 [28]. The associations were tested between exposure variables (sex (male or female), age (≤1 year and >1 year), breed (pure or mixed), free roaming (yes or no), ectoparasite infestation (yes or no), and the tri-monthly application of ectoparasiticides (yes or no)). Odds ratio (OR) and 95% confidence interval (95% CI) were calculated. The variables in the statistical likelihood ratio at *p* < 0.05 were considered statistically significant.

## 3. Results

### 3.1. Prevalence of CVBPs in Pet Dogs and Cats

Among the 210 pets (95 dogs and 115 cats), the proportion of reported CVBP infections was 10.5%. Among the 95 dogs, 6.3% (6/95) were found positive for *Ehrlichia*, while 1.1% (1/95) were positive for *Anaplasma*. Furthermore, one dog (1.1%) was co-infected with *Ehrlichia* and *Anaplasma*, while *Babesia*, *Bartonella*, *Hepatozoon*, *Mycoplasma,* and *Rickettsia* were not detected in any dogs. In cats, the prevalence of CVBPs was 13.9% (16/115), and *Mycoplasma* was detected in cats (11/115, 9.6%) at a higher proportion than *Rickettsia* (4.4%, 5/115), while *Anaplasma*, *Babesia*, *Bartonella*, *Ehrlichia*, and *Hepatozoon* were not detected in cats.

### 3.2. Genetic Characterization of CVBPs in Dogs and Cats

All 22 positive amplicons with CVBPs detected using PCR were submitted for sequence and genetic characterization based on comparisons with known sequences. Among the 11 cat samples positive for *Mycoplasma*, there were four samples presenting 99.4% identity with the sequence reported as *Mycoplasma haemofelis* (GenBank accession number MW633343), and seven samples were detected showing 99.6% identity with *Candidatus* Mycoplasma haemoninutum (GenBank accession number MW598402). All five obtained sequences of *Rickettsia* shared 99.2% identity with the published sequence of *Rickettsia felis* (accession number GQ385243). Regarding *Ehrlichia*, all six positive amplicons from dogs were confirmed as *E. canis* with 99.2% identity (GenBank accession number KU765198). The samples positive for *Anaplasma* had 97.1% identity with the reported sequence for *Anaplasma platys* (GenBank accession number CP046391).

### 3.3. Phylogenetic Analysis

**Figure 2:**
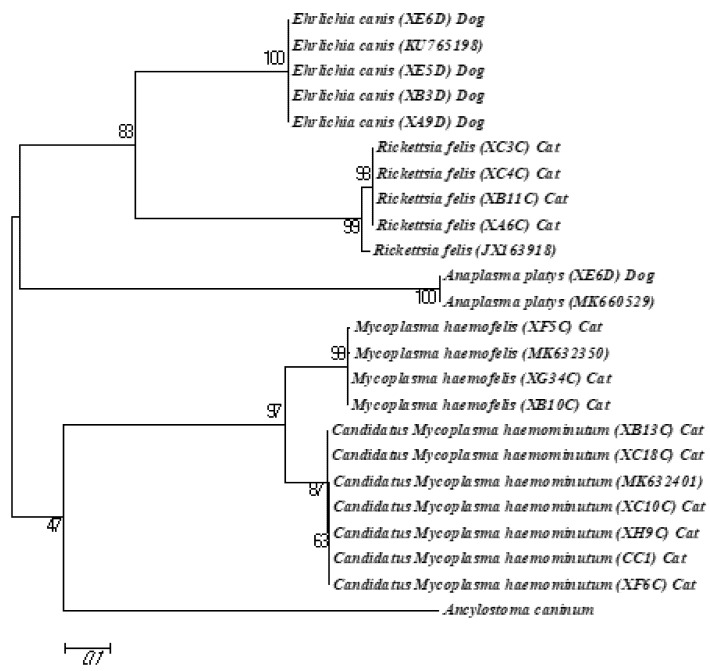
Phylogenetic tree of each CVBP sequence based on *gltA* gene (*Ehrlichia*), *ompB* gene (*Rickettsia*), *groESL* gene (*Anaplasma*), and 16S rRNA gene (*Mycoplasma*) using maximum likelihood method (Tamura–Nei parameter model). Numbers at each node represent the percentage occurrence of clades based on 1000 bootstrap replications of data, with *Ancylostoma caninum* provided as outgroup.

The phylogenetic analysis involving *A. platys*, *E. canis*, *R. felis*, *M. haemofelis*, and *Candidatus* Mycoplasma haemoninutum demonstrated that the variability between the sequences of these pathogens was low compared to those from other geographical regions. The *A. platys* and *E. canis* sequences were identical to the reference sequences (accession no. KU765198 and MK660529), whereas a low degree of genetic variability was observed in the *R. felis*, *M. haemofelis,* and *Candidatus* Mycoplasma haemoninutum sequences compared with their respective reference sequences (accession nos. JX163918, MK632350, and MK632401, respectively). All the isolates of each species formed separate clades, with a high bootstrap support (Figure 2).

### 3.4. Risk Factors Associated with CVBP Infections in Dogs and Cats in Khukhot City Municipality, Pathum Thani, Thailand

CVBP exposure risk was significantly associated with age group in both dogs and cats. In particular, young dogs (≤1 year) were more likely to be infected with CVBPs than adult dogs (OR 8.5, 95% CI 1.4–50.1, *p* = 0.006). Additionally, dogs regularly receiving ectoparasite prevention treatment were more likely to be infected with CVBPs than untreated dogs (OR 5.8, 95% CI 1.1–32.2, *p* = 0.025). However, there were no significant differences among other factors in this study (Table 2).

Regarding CVBP infections in cats, a significant variation was found between cat age groups. Specifically, adult cats (>1 year) were more likely to be infected with CVBPs than young cats (≤1 year) (OR 3.8, 95% CI 1.0–14.0, *p* = 0.038). However, there were no significant differences among the other risk factors for CVBP infections in cats (Table 3).

## 4. Discussion

CVBPs have been reported in several countries in Southeast Asia [29,30]. In addition, the current study established the presence of companion vector-borne Alphaproteobacteria (*Anaplasma* spp. and *Ehrlichia* spp.) in dogs, while, to date, there has been no published record of the prevalence of apicomplexan protozoan (*Babesia* spp. and *Hepatozoon* spp.). In the current study, the prevalence of *Ehrlichia* spp. infection in dogs (6.3%) was lower than the prevalence reported in another study of domestic dogs in Buriram province (36.7%) [18], but it was higher than that of another study in domestic dogs from Khon Kaen Province (reporting 3.0% prevalence) [19]. In addition, the prevalence of *Anaplasma* spp. (1.1%) in the current report was slightly lower than the equivalent in another report in Songkhla Province, Southern Thailand, with 3.9% prevalence [31]. These differences in the prevalence levels of *Ehrlichia* and *Anaplasma* infections in dogs could be due to various factors, such as differences in the study design, sample size, geographical location, vector population and exposure, and host immune status.

The current research revealed that young dogs had a higher chance of being infected with CVBPs than adult dogs, which corroborated another study of dogs in central Chile [32]. There are several potential reasons why young dogs may be more susceptible to CVBP infection compared to adult dogs. One possibility is that the development of the immune system in dogs is a gradual process, and young dogs may not have developed sufficient immunity to effectively fight off infections. Additionally, younger dogs may be more likely to engage in types of behavior that increase their exposure to ectoparasites.

As expected, dogs that had ectoparasites were more likely to be infected by CVBPs, most likely as a result of the increased chance of exposure to tick and flea bites, as ticks and fleas can be vectors of these pathogens [33]. In another study, dogs that did not receive proper hygienic care from their owners or antiparasitic treatments were more likely to be affected by CVBPs [34]; however, the current results differed from this, as dogs treated regularly with ectoparasiticides (spot-on or oral) were infected with CVBPs more than untreated dogs. Regarding the ectoparasite prevention and treatment in the current study, a common observation was that owners often delayed treatment until after the ectoparasite infestation had been detected. This could have resulted in a delay in treatment, during which time the dogs were exposed to CVBPs. It remains unclear whether the treatment was successful and it was a latent infection that had returned. Further research is needed to better understand the relationship between the timing of ectoparasite treatment, pathogen detection, and the risk of infection in dogs.

In cats, this study established the presence of flea-borne pathogens, such as *Mycoplasma* spp. and *Rickettsia* spp. The findings showed that the predominant pathogen was *Mycoplasma* spp., while *Rickettsia* spp. was reported less frequently. Another report commented on the association between *Mycoplasma haemofelis* and hematological findings [35]. However, because of the small number of hemoplasma-infected samples, there was no evidence in the current investigation that hemoplasma species were linked with hematological change.

Age group was the major factor variable associated with CVBP infections. The study also established that cats that could roam freely were more likely to be infected than those restricted to an in-house environment. This was corroborated by other publications that made the same observation, i.e., cats that could roam outside had reportedly increased chances of coming into contact with wild animals, which were thought to be a cause of several infectious diseases [36]. Furthermore, male cats have a reportedly higher risk of CVBP infection according to the majority of prevalence studies carried out worldwide [35]; however, this was not observed in the current study.

Given that *R. felis* is an acknowledged zoonotic pathogen [37,38,39], future recommendations should be developed for successful chemoprophylaxis, regular examinations of domestic animals, and efficient ectoparasite control strategies. Furthermore, the characterization of these zoonotic pathogens from companion animals and humans living in the same household or shared environment is necessary to determine the human–animal transmission.

## 5. Conclusions

The current report demonstrated a low occurrence of CVBPs in apparently healthy pet animals (dogs and cats) in Pathum Thani province, Thailand. Nevertheless, the updated data in this research illustrated an overall picture of CVBPs affecting apparently healthy pet animals, which is an important issue for animal and public health, because some species considered were zoonotic pathogens. In addition, the current research demonstrated the need for further epidemiological studies of CVBPs in companion dogs and cats with greater sample sizes to determine the extent to which these pets serve as significant reservoir hosts for infections with vector-borne diseases in Thailand.

## Figures and Tables

**Table 1:** Primers for characterization of CVBPs in pet animals.

Pathogen	Target Genes	Primer Sequences (5′–3′)	Amplicon Size (bp)	References
*Babesia*	18S rRNA	GTTTCTGMCCCATCAG CTGTATTGTTATTTCTTGTCACTACCTC	422–440	[21]
*Anaplasma*	*groEL*	AAGGCGAAAGAAGCAGTCTTA CATAGTCTGAAGTGGAGGAC	724	[22]
*Ehrlichia*	*gltA*	TTATCTGTTTATGTTATATAAGC CAGTACCTATGCATATCAATCC	1251	[23]
*Hepatozoon*	18S rRNA	ATACATGAGCAAAATCTCAAC CTTATTATTCCATGCTGCAG	666	[24]
*Bartonella*	*gltA*	GGGGACCAGCTCATGGTGG AATGCAAAAAGAACAGTAACA	379	[25]
*Mycoplasma*	16S rRNA	ATACGGCCCATATTCCTACG TGCTCCACCACTTGTTCA	595–618	[26]
*Rickettsia*	*ompB*	CGACGTTAACG GTTTCTCATTCT ACCGGTTTCTTTGT AGTTTTCGTC	252	[27]

*groESL*, heat-shock protein gene; *gltA*, citrate synthase gene; *ompB*, outer-membrane protein B.

**Table 2:** Risk factors associated with CVBPs in apparently healthy pet dogs in Pathum Thani, Thailand.

Factor	Number of Dogs	Number of Positives (%)	Chi-Square *χ*^2^	Odds Ratio (95% CI)	*p*-Value
Sex			0.43		0.510
Male	44	2 (4.6)		1.00	
Female	51	4 (7.8)		0.6 (0.1–3.2)	
Age			7.39		0.006
≤1 year	21	4 (19.0)		8.5 (1.4–50.1)	
>1 year	74	2 (2.7)		1.00	
Breed			NA		NA
Pure	22	0		NA	
Mixed	73	6 (8.2)		NA	
Free-roaming			0.05		0.830
Yes	28	2 (7.1)		0.8 (0.1–4.8)	
No	67	4 (6.0)		1.00	
Ectoparasite infestation			0.20		0.656
Yes	72	5 (6.9)		0.6 (0.1–5.5)	
No	23	1 (4.4)		1.00	
Tri-monthly application of ectoparasiticides			5.03		0.025
Yes	16	3 (18.6)		5.8 (1.1–32.2)	
No	79	3 (3.8)		1.00	

NA: not applicable.

**Table 3:** Risk factors associated with CVBPs in apparently healthy pet cats in Pathum Thani, Thailand.

Factor	Number of Cats	Number of Positives (%)	Chi-Square *χ*^2^	Odds Ratio (95% CI)	*p*-Value
Sex			0.56		0.453
Male	34	6 (17.6)		1.00	
Female	81	10 (12.3)		0.7 (0.2–2.0)	
Age			4.33		0.038
≤1 year	49	3 (6.1)		1.00	
>1 year	66	13 (19.7)		3.8 (1.0–14.0)	
Breed			0.43		0.514
Pure	4	1 (25.0)		1.00	
Mixed	111	15 (13.5)		0.5 (0.1–4.8)	
Free-roaming			1.21		0.271
Yes	91	11 (12.1)		1.00	
No	24	5 (20.8)		1.9 (0.6–6.2)	
Ectoparasite infestation			1.07		0.300
No	98	15 (15.3)		1.00	
Yes	17	1 (5.9)		0.3 (0.1–2.8)	
Tri-monthly application of ectoparasiticides			0.61		0.435
No	101	15 (14.9)		1.00	
Yes	14	1 (7.1)		0.4 (0.1–3.3)	

## Data Availability

Not applicable.
